# The Key Roles of Negative Pressure Breathing and Exercise in the Development of Interstitial Pulmonary Edema in Professional Male SCUBA Divers

**DOI:** 10.1186/s40798-017-0116-x

**Published:** 2018-01-03

**Authors:** Olivier Castagna, Jacques Regnard, Emmanuel Gempp, Pierre Louge, François Xavier Brocq, Bruno Schmid, Anne-Virginie Desruelle, Valentin Crunel, Adrien Maurin, Romain Chopard, David Hunter MacIver

**Affiliations:** 1 Underwater Research Team (ERRSO) from the Military Biomedical Research Institute (IRBA), Toulon, France; 2 Laboratory of Human Motricity, Education Sport and Health, LAMHESS (EA 6312), Toulon, France; 3 EA3920, University Bourgogne Franche-Comté and University Hospitals, Besançon, France; 4 Department of Hyperbaric Medicine, HIA St Anne Military Hospital, Toulon, France; 5 French Navy Diving School, Toulon, France; 6 Department of Cardiology, HIA St Anne Military Hospital, Toulon, France; 7 Department of Cardiology EA3920, Franche Comté University and University Hospital, Besançon, France; 8 Biological Physics Group, University of Manchester, Manchester, UK; 9 Musgrove Park, Taunton & Somerset Hospital, Taunton, UK

**Keywords:** Atrial natriuretic peptide, Echocardiography, Exercise, Hydrostatic transrespiratory pressure, Immersion pulmonary edema, Inspiratory breathing effort, Lung ultrasonography, Negative pressure breathing, Right heart preload, Work of breathing

## Abstract

**Background:**

Immersion pulmonary edema is potentially a catastrophic condition; however, the pathophysiological mechanisms are ill-defined. This study assessed the individual and combined effects of exertion and negative pressure breathing on the cardiovascular system during the development of pulmonary edema in SCUBA divers.

**Methods:**

Sixteen male professional SCUBA divers performed four SCUBA dives in a freshwater pool at 1 m depth while breathing air at either a positive or negative pressure both at rest or with exercise. Echocardiography and lung ultrasound were used to assess the cardiovascular changes and lung comet score (a measure of interstitial pulmonary edema).

**Results:**

The ultrasound lung comet score was 0 following both the dives at rest regardless of breathing pressure. Following exercise, the mean comet score rose to 4.2 with positive pressure breathing and increased to 15.1 with negative pressure breathing. The development of interstitial pulmonary edema was significantly related to inferior vena cava diameter, right atrial area, tricuspid annular plane systolic excursion, right ventricular fractional area change, and pulmonary artery pressure. Exercise combined with negative pressure breathing induced the greatest changes in these cardiovascular indices and lung comet score.

**Conclusions:**

A diver using negative pressure breathing while exercising is at greatest risk of developing interstitial pulmonary edema. The development of immersion pulmonary edema is closely related to hemodynamic changes in the right but not the left ventricle. Our findings have important implications for divers and understanding the mechanisms of pulmonary edema in other clinical settings.

## Key points

- The exercise-induced increase in tidal volume during immersion elevates right heart preload, triggering a right to left ventricular imbalance and lung congestion.
- Exercising with negative pressure breathing further increases the inspiratory work of breathing, right ventricle loading, right to left heart imbalance, and rate of interstitial lung water accumulation.
- Positive pressure breathing decreases cardiovascular changes and pulmonary edema during immersion with exercise.
- Plasma levels of atrial natriuretic peptide increase with inspiratory work and correlates with lung comet scores.
- An altered right to left heart imbalance provokes the development of immersion pulmonary edema when inspiratory work is high, e.g., during swimming at high intensity level or SCUBA diving with negative pressure breathing setting.

## Background

Immersion pulmonary edema (IPE) is accompanied by the onset of dyspnea while diving or swimming. IPE may be accompanied by cough, hemoptysis, and severe hypoxemia and can result in death [1–3]. Resting and normobaric oxygen therapy usually results in rapid relief of symptoms without sequelae. IPE can occur in both young athletes as well as older subjects especially if cardiovascular co-morbidities are present [4].

In healthy subjects, the main predisposing factors to IPE [5] are cold water [3, 6] and exertion [7, 8]. Other predisposing factors are age > 50 years, hypertension, and left heart disease [4]. The condition has also been reported in highly fit subjects such as military swimmers and triathletes [1–3, 9]. IPE symptoms can vary in severity [10], and the accumulation of interstitial pulmonary edema without overt symptoms is common after normal diving without significant exertional effort [11]. Moderate fin swimming exercise leads to increasing interstitial pulmonary edema [12]. The rise in transmural pulmonary capillary pressure causes transudation initially into the interstitial tissues, [13] as evidenced by the appearance of ultrasound “lung comet tails,” [14] before reaching the alveolar air spaces [15].

We recently reported that 30-min finning during SCUBA air dive at shallow depth resulted in interstitial pulmonary edema in 11 of 15 study subjects [12]. An increased preload and a right-left heart imbalance correlated with the accumulation of extravascular lung water (EVLW). We showed that changes in right ventricular physiology, rather than left ventricular indices, correlated with the development of interstitial pulmonary edema. The severity of interstitial pulmonary edema was significantly correlated with measures of increased right ventricular filling, right ventricular area change (a surrogate of right ventricle ejection fraction), and pulmonary artery pressure. Left ventricle ejection fraction (LVEF) and left ventricle stroke volume (LVSV) did not change. We concluded that the imbalance between right and left heart stroke volumes during effort is central to the development of immersion pulmonary edema.

The effort of breathing is greater during immersion than on land because the external hydrostatic pressure creates a greater transpulmonary pressure difference [16, 17] and results in a more negative breathing pressure (NPB). NPB is present during swimming because the airway pressure is lower than the hydrostatic pressure surrounding the thorax and the abdomen [8, 10, 16]. During diving, when the open diving regulator is held at the mouth, NPB occurs when changing from prone to upright posture as arises when returning towards surface [16, 18]. During NPB, the inspiratory effort increases, generates a lower pleural and airway pressures, and results in greater tidal swings in thoracic pressure while preserving the airway flow rate. Conversely, positive pressure breathing (PPB) or inspiratory assistance decreases the transpulmonary pressure gradient [19, 20]. Although an increased inspiratory effort is recognized as a respiratory burden to divers, [17, 21, 22] no previous study, to our knowledge, has directly assessed the cardiovascular and pulmonary effect of airway pressure during immersed exercise.

NPB increases the transmural hydrostatic pressure difference between the lumen of the lung capillaries and interstitial fluid in bronchial bundles and alveoli. NPB also alters cardiac function [23] and can trigger acute pulmonary edema on land [2]. The increase in capillary transmural pressure is considered a key factor in the mechanism of IPE occurrence as expected from the Starling equation [24]. Indeed, on land, NPB-induced interstitial pulmonary edema is similar to that observed during finning exercise, i.e., an increase in right ventricular function without an increase in the left ventricle function. We proposed, therefore, that the effects of immersion and NPB might individually alter heart function and amplify the risks of developing IPE.

During immersion, the rise in peripheral venous return to the heart induces a release of atrial natriuretic peptide (ANP) [25]. On land, NPB increases transmural pressure of atrial wall, and triggers ANP release [26]. Furthermore, an elevated ANP might encourage the development of IPE because ANP increases capillary permeability [27].

We, therefore, designed a study to investigate both the independent and combined effects of (i) inspiratory breathing pressure setting and (ii) exercising on cardiovascular physiology and the development of interstitial pulmonary edema.

## Methods

Sixteen professional male SCUBA divers were recruited. All volunteers were healthy and non-smokers and had no history of cardiovascular or pulmonary disease. Each gave written informed consent for participation in this study. The characteristics of these subjects were as follows (mean ± SD): age 34.4 ± 12.1 year, height 1.84 ± 0.12 m, and body weight 68.4 ± 7.7 kg. All experimental procedures were conducted in line with the Declaration of Helsinki, and the study protocol was approved by the local Ethics Committee (Comité de Protection des Personnes-CPP Sud Méditerranée V, ref 16.077). The methods and potential hazards were explained to participants in detail before beginning the experiments.

Each diver completed four 30-min air-breathing dives in a 29 °C freshwater pool, at shallow depth (≈ 1 m). The four sessions were randomly allocated and 72 h apart. The divers refrained from exercise and any dive for 24 h before each experimental session. On each dive, they wore trunks without a wetsuit and used the same closed-circuit rebreather SCUBA setting (Triton®, MS3, Tourves, France) and remained in prone position.

**Fig. 1:**
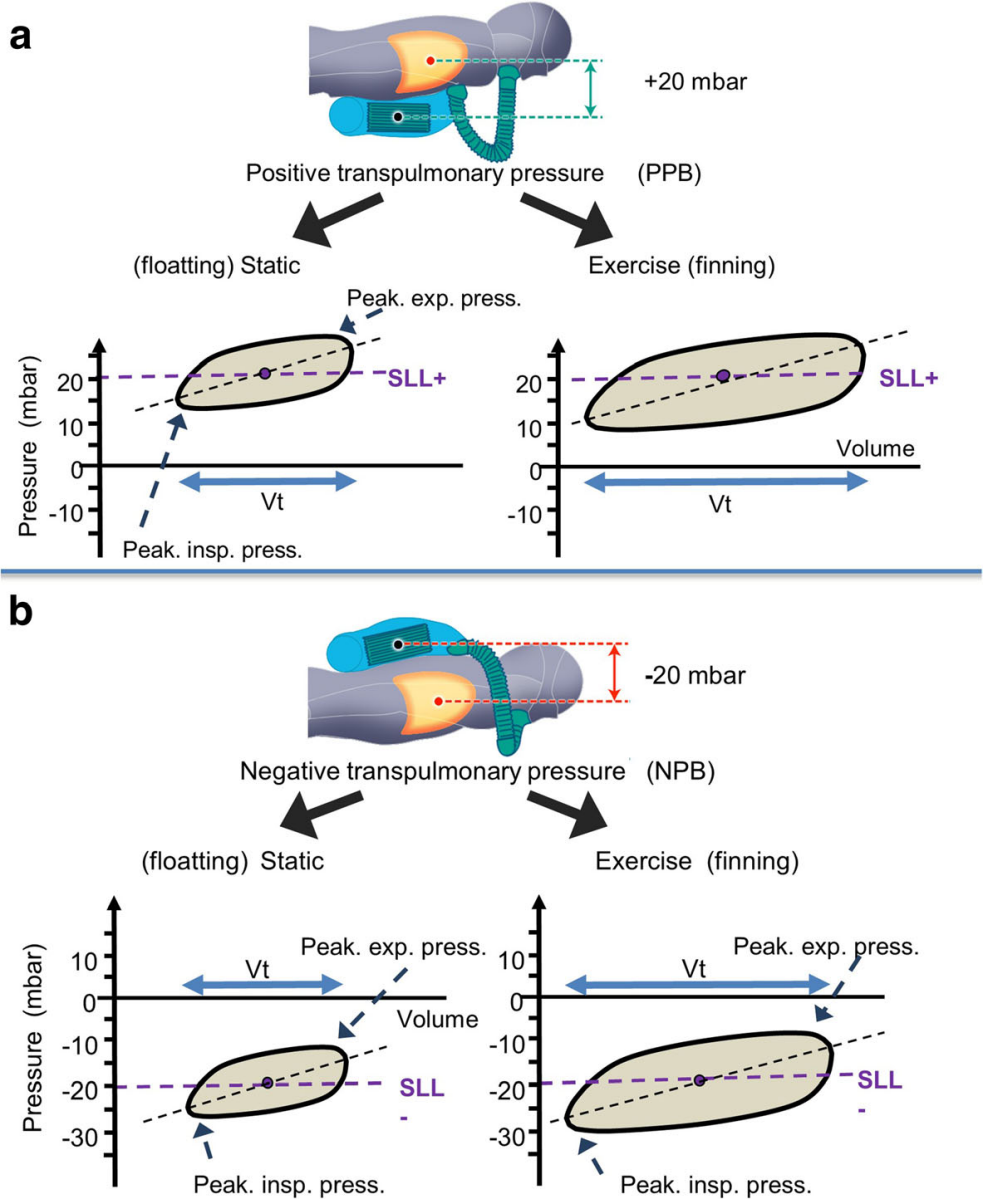
Tidal volume loop during each dive condition in one diver. A positive transpulmonary pressure gradient (or positive static lung load: SLL+) is set when the rebreather is worn anteriorly (on the abdomen) by the diver in prone position (**a**). A positive pressure breathing (PPB) condition is created. Conversely, when the rebreather is worn posteriorly (**b**), the transpulmonary pressure gradient is negative in the prone position (negative static lung load, SLL−), and the diver is in condition of negative pressure breathing (NPB). In each condition, the diver completed two 30-min dives, one simply statically floating (static), and one with continuous fin swimming (exercise). Examples of tidal pressure-volume loops are sketched during both static and exercise in each PPB and NPB condition. The dashed lines indicate the SLL level in each condition. Peak insp. press., peak inspiratory pressure during; peak expir. press., peak expiratory pressure. Of note, in each PPB and NPB, Vt lengthening carried the main rest to exercise change, while pressure ranges were very similar during static and exercise dives

The static conditions (*Static*) consisted in floating at rest, breathing with a positive pressure when the rebreather was attached anteriorly (*StPPB*), and with a negative pressure when the rebreather was attached posteriorly (*StNPB*) (Fig. 1a, b). During exercise (*Exercise*), subjects were asked to fin swim throughout the 30 min of immersion while maintaining a heart rate (HR) of 110 ± 10 bpm (monitored with a Polar® V800, Finland) to achieve constant moderate work intensity.

Prior to immersion, resting cardiovascular indices and the absence of EVLW were assessed based on cardiopulmonary ultrasonography. During immersion, ventilatory flow and pressure were continuously measured in the mouthpiece. Transthoracic echocardiography was performed immediately after exertion while still submerged. Lung ultrasound was used to assess for the presence of EVLW, and a single-breath gas transfer capacity of the lung (TLCO) was measured. Pulmonary artery pressure was assessed from the tricuspid regurgitant jet. Two venous blood samples were taken from the antecubital vein before and immediately after immersion.

### Functional Assessments

#### Pulmonary Function

TLCO was measured prior to immersion and 20 min after emersion using a computerized Quark Pulmonary Function Test device (Cosmed, Rome, Italy).

#### Cardiovascular Indices

Ultrasonographic examinations were performed using a *Vivid i* device (General Electric, Milwaukee, USA) with a 1.3–4.0-MHz array transducer. Cardiac chamber sizes were assessed according to recent guidelines [25, 26].

The same device was used to monitor EVLW based on the number of B-lines or ultrasound lung comet tails (ULC) counted on images [27–29] using the protocol recommended by Gargani et al. [28]. Individual ULC score was the sum of each B-lines assessed in all scanning sites.

#### Biology

Venous blood samples were drawn to assay plasma concentrations of adrenaline and noradrenaline with high-pressure liquid chromatography coupled with electrochemical detection. The plasma concentrations of ANP and BNP were also measured as the stoichiometric pro-peptides whose longer half-lives provide a good assessment of the cumulated release over a 30-min period (respectively proANP—using the enzymatic immunoassay kit Nt-proANP: Biomedica, Wien, Austria, and proBNP—using the Elecsys Nt-proBNP immunoassay kit: Roche Diagnostics, Indianapolis, USA).

The work of breathing (WOB) was assessed for every breath, using a bespoke electronic pneumo-baro-tachograph placed between the SCUBA regulator and the mouthpiece. Assessing the tidal pressure cycle at the mouth allows calculating the WOB (Joules) from the area of the pressure-Vt loop. The WOB/Vt defines the pressure required to perform one unit (L) tidal volume as suggested by Warkander et al. [29]. The cumulative WOB is the total work done performing the breathing cycle during each of the 30-min sessions (cWOB).

### Statistics

Statistical analysis was performed with the Prism 6 software (GraphPad Software, La Jolla California USA). Each subject served as his own control. Data distribution was assessed using a Kolmogorov–Smirnov test. For values obtained at four-time points, two-way repeated-measures analysis of variance (positive or negative pressure breathing, and rest or fin exercising immersion) was performed (with the post hoc Holm–Sidak test) when the data were normally distributed. For non-normally distributed data, comparisons relied on a Friedman’s test and on the post hoc dichotomous comparisons with a Dunn’s test. Correlations between ULC score and cardiac function were assessed using Pearson’s test. The same test was used to assess correlations between cWOB and cardiac indices. Differences between groups were considered statistically significant at *p* < 0.05. All values are expressed as mean ± SD.

## Results

### Heart Rate and Ventilatory Status at the End of Each Session (Table 1)

**Fig. 2:**
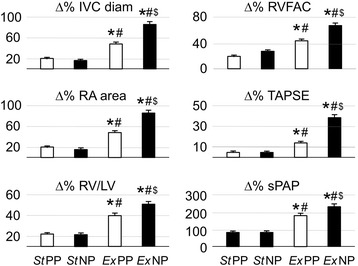
Percent changes in parameters of right cardiac function after 30-min dive in each combination of pressure breathing and physical activity. StPP, static dive with positive transpulmonary pressure; StNP, static dive with negative transpulmonary pressure; ExPP, continuous finning dive with positive transpulmonary pressure; ExNP, continuous finning dive with negative transpulmonary pressure; IVC diam, diameter of inferior vena cava; RA area, right atrial area; RV/LV, ratio of right to left ventricle end-diastolic area; RVFAC, right ventricle fractional area change; TAPSE tricuspid annular plane systolic excursion; sPAP, systolic pulmonary arterial pressure. *p < 0.05 significant difference between ExPP and StPP or ExNP and StNP; ^#^*p* < 0.05 significant difference between ExPP and StNP or ExNP and StPP; ^$^*p* < 0.05 significant difference between ExNP and ExPP. Two-way analysis of variance (ANOVA) with repeated-measures and the post hoc Holm–Sidak test were used to compare the four conditions in each variable

Mean heart rate was the same 110 min^−1^ after 30-min static (resting) dives performed in either positive and negative pressure breathing condition in line with the protocol. Tidal volume and breathing rate were similar during both static and exercise dives and independent of breathing pressure (Fig. 2a, b). On average, exercise tidal volume was almost three times the static value while the breathing rate almost doubled with exercise. Minute ventilation was the same in the two static sessions and during the two exercise sessions and about five times higher during exercise compared with the static dives (*p* < 0.001). As expected, the peak inspiratory and expiratory pressures were dependent on transpulmonary pressure difference (i.e., the static lung load) both without and with exercise. Peak inspiratory and expiratory pressures were slightly but significantly greater at the end of exercise compared with rest sessions in both PPB and NPB groups. The tidal and cumulated inspiratory work of breathing were higher following the static NPB than PPB dive. Exercise increased the inspiratory WOB twofold with PPB and threefold with NPB. The cumulated inspiratory work of breathing over 30 min was increased fourfold with PPB and fivefold with NPB.

**Table 1:** In-water heart rate and ventilatory variables at the end of the four dive sessions

	Static		Fin exercise	
	PPB (A)	NPB (B)	PPB (C)	NPB (D)
Heart rate (bpm)	58.9 ± 4.9	50.1 ± 4.7	111.6 ± 5.6^a^	109.8 ± 45.5^a^
Tidal volume (L)	1.3 ± 0.1	1.3 ± 0.2	3.5 ± 0.2^a^	3.5 ± 0.2^a^
Minute ventilation (L min^−1^)	8.2 ± 2.2	8.2 ± 2.7	41.3 ± 9.7^a^	40.3 ± 9.6^a^
Breathing frequency (min^−1^)	6.4 ± 1.4	6.3 ± 1.3	11.8 ± 2.1^a^	11.5 ± 2.2^a^
Peak inspiratory pressure (mbar)	14.9 ± 1.8	− 25.4 ± 1.7^b^	12.7 ± 1.6	− 27.6 ± 1.58^b^
Peak expiratory pressure (mbar)	22.8 ± 1.2	− 18.6 ± 1.5^b^	25.2 ± 1.1	− 15.3 ± 1.9^b^
Static lung load (mbar)	18.8 ± 1.5	− 21.9 ± 1.3^b^	18.9 ± 1.3	− 21.4 ± 1.1^b^
WOB *insp*. (J)	− 1.9 ± 0.3	3.2 ± 0.7^b^	− 4.4 ± 0.8	9.5 ± 0.9^ab^
WOB/Vt *insp*. (J L^−1^)	− 1.5 ± 0.2	2.5 ± 0.2^b^	− 1.3 ± 0.2	2.8 ± 0.2^b^
cWOB *insp.* (J)	− 376 ± 140	+636 ± 244^b^	− 1615 ± 560^a^	+ 3345 ± 868^ab^
Ultrasound lung comet	0	0	4.2 ± 2.3^a^	15.1 ± 15.3^ab^

Two-way analysis of variance (ANOVA) with repeated-measures and the post hoc Holm–Sidak test were used to compare values in the four conditions for each variable.

*Abbreviations: Static* dive session simply floating without physical activity, *Fin exercise* dive with continuous fin swimming, *PPB* positive pressure breathing condition caused by positive transpulmonary hydrostatic difference or positive static lung load, *NPB* negative pressure breathing condition caused by negative transpulmonary hydrostatic difference or negative static lung load, *SLL* static lung load, *WOB insp.* breathing work for one tidal inspiration, *WOB/Vt insp.* one-cycle inspiratory work of breathing per volume unit, *cWOB* work of breathing cumulated over the 30-min session

^a^Exercise different from static dive

^b^Static-NPB different from static-PPB, or exercise-NPB different from exercise-PPB

No lung comets were seen prior the dives or after resting dives, whereas there was an average of 4.2 comets following exercise with PPB and 15.1 comets with NPB (*p* < 0.05).

### Effects on Venous Return, Right Heart Function, and Pulmonary Artery Pressure (Table 2 and Fig. 2)

Pre-dive values were no different between the four sessions, in any variable: diameter of inferior vena cava (IVCdiam), right atria area (RAa), right ventricle end-diastolic area (RVEDa), tricuspid annular plane systolic excursion (TAPSE), and systolic pulmonary artery pressure (sPAP). Pulmonary artery pressure was calculated in 11 of the 16 subjects. At the end of static dives, IVCdiam and RAa were significantly increased by approximately 20% as compared to pre-dive values, and sPAP by 80%, in both PPB and NPB conditions. The end-of-dive TAPSE increased significantly following NPB session (*p* < 0.001). In summary, 30-min static dive triggered a rise in right heart preload and pulmonary arterial pressure compared with baseline values. In addition, NPB caused a greater RV contractility (TAPSE) compared with PPB (*p* < 0.001).

**Table 2:** Cardiac variables assessed through transthoracic echography in-water at start and end of each dive session

	Static PPB		Static NPB		Exercise PPB		Exercise NPB	
	Pre-dive (A)	Post-dive (A′)	Pre-dive (B)	Post-dive (B′)	Pre-dive (C)	Post-dive (C′)	Pre-dive (D)	Post-dive (D′)
Right heart								
IVC diameter (cm)	1.7 ± 0.1	1.8 ± 0.1^a^	1.7 ± 0.1	1.8 ± 0.1^a^	1.7 ± 0.2	2.5 ± 0.7^ab^	1.7 ± 0.1	3.2 ± 0.5^abc^
RA area (cm^2^)	12.4 ± 1.5	14.8 ± 2.5^a^	12.2 ± 1.6	14.2 ± 2.3^a^	12.3 ± 1.8	18.2 ± 2.7^ab^	11.9 ± 1.5	22.1 ± 3.0^abc^
RVED area (cm^2^)	19.1 ± 2.5	22.9 ± 3.1	18.9 ± 2.3	21.9 ± 2.6	18.9 ± 2.4	25.9 ± 3.2^ab^	19.7 ± 2.4	28.2 ± 4.5^abc^
RVES area (cm^2^)	11.9 ± 2.7	11.8 ± 2.6	12.2 ± 2.3	11.9 ± 3.2	12.8 ± 2.9	12.4 ± 2.4	12.2 ± 1.9	12.7 ± 2.9
RVFAC (%)	37.6 ± 10	45.1 ± 8.4	25.1 ± 6.9	45.3 ± 7.2	35.9 ± 9.2	52.1 ± 7.7^ab^	33.4 ± 9.7	55.7 ± 7.3^ab^
TAPSE (mm)	20.7 ± 1.2	21.6 ± 1.4	20.8 ± 1.2	21.8 ± 1.2^a^	21.5 ± 1.1	24.4 ± 1.5^ab^	20.6 ± 1.1	28.6 ± 1.7^abc^
_S_PAP (mmHg)	7.6 ± 1.1	13.7 ± 2.5^a^	7.2 ± 1.2	13.1 ± 2.2^a^	7.2 ± 0.7	20.2 ± 2.1^ab^	7.5 ± 0.7	24.5 ± 2.9^abc^
Left heart								
LA area (cm^2^)	13.5 ± 1.2	14.9 ± 1.3^a^	13.1 ± 1.0	14.5 ± 1.1^a^	13.4 ± 1.6	15.1 ± 1.7^a^	13.7 ± 2.2	16.4 ± 2.8^a^
LVEF (%)	63.7 ± 2.6	65.1 ± 2.4	64.7 ± 2.6	64.67 ± 2.1	63.6 ± 3.1	64.9 ± 2.5	65.1 ± 2.1	65.9 ± 1.7
SV (mL)	74.8 ± 3.1	75.1 ± 3.1	75.8 ± 2.6	73.1 ± 2.1	74.6 ± 2.3	74.1 ± 2.7	75.6 ± 3.5	75.5 ± 2.4
HR (bpm)	58.8 ± 4.9	50.1 ± 4.6^a^	61.4 ± 4.7	51.3 ± 4.4^a^	57.4 ± 6.1	111.6 ± 6.1^ab^	60.1 ± 5.5	109.8 ± 5.5^ab^
CO (L m^−1^)	4.4 ± 0.6	3.8 ± 0.5^a^	4.7 ± 0.5	3.7 ± 0.4^a^	4.3 ± 0.6	8.3 ± 0.7^ab^	4.6 ± 0.6	8.3 ± 0.7^ab^
LVED area (cm^2^)	33.2 ± 3.1	32.6 ± 4.1	32.2 ± 3.7	35.4 ± 3.1^a^	33.3 ± 4.3	33.1 ± 3.7	32.9 ± 3.7	32.7 ± 2.7^b^
LVES area (cm^2^)	18.2 ± 1.5	17.9 ± 1.4	17.6 ± 1.2	17.8 ± 1.4	18.2 ± 1.4	17.7 ± 1.5	17.3 ± 1.2	18.0 ± 1.4
RV/LV area (%)	57.2 ± 2.8	67.3 ± 2.6^a^	58.8 ± 2.2	61.7 ± 2.9^a^	57.0 ± 2.8	78.5 ± 4.5^ab^	56.8 ± 1.9	85.9 ± 8.3^ab^
E (m s^−1^)	0.79 ± 0.05	0.82 ± 0.04	0.79 ± 0.06	0.82 ± 0.04	0.78 ± 0.06	0.85 ± 0.04	0.78 ± 0.06	0.88 ± 0.05
A (m s^−1^)	0.54 ± 0.13	0.48 ± 0.06^a^	0.52 ± 0.10	0.49 ± 0.05^a^	0.54 ± 0.11	0.45 ± 0.07^ab^	0.53 ± 0.12	0.41 ± 0.07^abc^
EDT (ms)	223 ± 11.6	201 ± 11.3^a^	225 ± 12.5	201 ± 14.3^a^	223 ± 12.3	192 ± 11.3^ab^	223 ± 11.1	184 ± 10.2^abc^
*E*/*A*	1.53 ± 0.31	1.75 ± 0.13^a^	1.56 ± 0.20	1.67 ± 0.09^a^	1.49 ± 0.22	1.92 ± 0.22^ab^	1.53 ± 0.24	2.18 ± 0.25^ab^

Cardiac dimensions and functional parameters were determined at start and end of each 30-min dive. Time- and condition-linked differences in each variable were assessed using two-way repeated-measures analysis of variance (with the post hoc test).

*Static PPB* static (rest) dive with positive pressure breathing setting, *Static NPB* static dive with negative pressure breathing setting, *Exercise PPB and Exercise NPB* exercises dives with respectively positive and negative pressure breathing conditions, *IVC diameter* inferior vena cava diameter, *RA area* right atrial area, *RVED area* right ventricle end-diastolic area, *RVES area* right ventricle end-systolic area, *RVFAC* right ventricle fractional area change, *TAPSE* tricuspid annular plane systolic excursion, *sPAP* systolic pulmonary arterial pressure, *LA area* left atrial area, *LVEF* left ventricle ejection fraction, *SV* left stroke volume, *HR* heart rate, *CO* cardiac output, *LVED area* left ventricle end-diastolic area, *LVES area* left ventricle end-systolic area, *RV/LV* ratio of right to left ventricles end-diastolic area, *E* peak early diastolic left ventricular filling velocity, *A* late diastolic left ventricular filling velocity, *EDT* E peak deceleration time (early left ventricular filling deceleration time), *E*/*A* ratio of *E* to *A* velocities

^a^Post-dive different from pre-dive in the same session

^b^Post-exercise different from post-static counterpart (similar transpulmonary pressure breathing)

^c^Post-exercise NPB different from post-exercise PPB

At the end of exercise dives, the values of IVCdiam, RAa, RVEDa, TAPSE, and sPAP were all higher than their pre-dive counterparts and substantially higher than their static sessions. PPB during exercise markedly increased venous return, right heart preload, right ventricle contractility, and pulmonary artery pressure compared with the resting dives. This confirmed the changes observed in our previous report where divers used an open-circuit breathing device in open water [12]. Performing an identical exercise level with NPB amplified these changes in venous return and right heart preload, triggering the highest TAPSE values and a more than doubling sPAP as compared to pre-dive assessment (*p* < 0.0001). These results show a stepwise increasing loading of right heart and pulmonary vascular bed through (1) resting immersion, (2) PPB with exercise, and (3) NPB with exercise.

### Effects on Left Heart Function (Table 2)

There were no differences between the four pre-dive values in left atrial area, left ventricular ejection fraction, stroke volume, heart rate, end-diastolic and end-systolic areas of left ventricle, ratio of right to left ventricle areas, early and E-wave peak velocity and E-wave deceleration time. Heart rate and cardiac output were lower at the end of both PPB and NPB static dives than pre-dive (*p* < 0.001). Left atrial area was moderately increased after both static PPB and NPB dives (*p* < 0.001). Left ventricle end-diastolic area increased after the NBP dive (*p* < 0.001), whereas end-systolic area was unchanged after both dives. The early filling velocity (*E*) was higher after the NPB dive (*p* < 0.035). The late filling velocity (*A*) was lower after the PPB dive (*p* < 0.024). Both the PPB and NPB dives increased the *E*/*A* ratio (*p* < 0.042), decreased the early deceleration time (*p* < 0.0001), and increased the RV/LV area ratio (*p* < 0.015). Exercise dives almost doubled heart rate and cardiac output in both PPB and NPB (*p* < 0.0001), without a change in left ventricular stroke volume.

### Plasma Concentrations of Catecholamines and Natriuretic Peptides and Lung Transfer of Carbon Monoxide (Table 3)

Adrenaline levels decreased after the two static dives (*p* < 0.0001) and increased after the exercise dives (*p* < 0.0001). Plasma noradrenaline was unchanged by the two static dives but was markedly increased after the exercise dives (*p* < 0.0001).

**Table 3:** Plasma concentrations of Nt-proANP, Nt-proBNP, adrenaline, and noradrenaline before and after dives

	Static PPB		Static NPB		Exercise PPB		Exercise NPB	
	Pre-dive (A)	Post-dive (A′)	Pre-dive (B)	Post-dive (B′)	Pre-dive (C)	Post-dive (C′)	Pre-dive (D)	Post-dive (D′)
Hormone								
Adrenaline (pg mL^−1^)	43.2 ± 2.3	33.3 ± 2.2^a^	43.9 ± 1.9	33.5 ± 2.1^a^	44.8 ± 1.5	76.5 ± 5.5^ac^	44.3 ± 2.1	76.2 ± 5.6^ac^
Noradrenaline (pg mL^−1^)	305 ± 45	302 ± 35	291 ± 64	291 ± 73	295 ± 62	690 ± 49^abc^	289 ± 52	715 ± 57^ac^
Nt-proANP(nmol L^−1^)	0.55 ± 0.21	1.89 ± 0.27^a^	0.41 ± 0.17	1.73 ± 0.27^a^	0.45 ± 0.19	2.63 ± 0.26^ac^	0.47 ± 0.23	4.57 ± 0.28^abc^
Nt-proBNP (pmol L^−1^)	5.72 ± 0.62	6.28 ± 0.60	6.08 ± 0.72	5.89 ± 0.60	6.09 ± 0.64	5.72 ± 0.52	5.89 ± 0.65	5.94 ± 0.58
DLCO								
DLCO (mL min^−1^ mm^−1^ Hg^−1^)	35.2 ± 2.8	35.4 ± 3.4	35.1 ± 4.1	35.2 ± 3.8	35.6 ± 3.5	33.5 ± 4.3^a^	35.4 ± 3.8	31.1 ± 4.2^abc^
DLCO/*V*_A_ (mL min^−1^ mm^−1^ g^−1^ L^−1^)	4.8 ± 0.4	4.5 ± 0.5	4.7 ± 0.5	4.7 ± 0.4	4.7 ± 0.5	4.1 ± 0.3^a^	4.7 ± 0.5	3.9 ± 0.3^ab^

Plasma concentration was determined before and after each dive. Time-and condition-linked differences in each variable were assessed using two-way repeated-measures analysis of variance (with the post hoc test).

*Static PPB* static (rest) dive with positive pressure breathing setting, *Static NPB* static dive with negative pressure breathing setting, *Exercise PPB and Exercise NPB* exercises dives with respectively positive and negative pressure breathing conditions, *Nt-proANP* N-terminal fraction of pro-atrial natriuretic peptide, *Nt-proBNP* N-terminal fraction of pro-brain natriuretic peptide, *TLCO* lung transfer factor for carbon monoxide, *TLCO/VA* ratio of lung transfer factor for carbon monoxide to alveolar volume assessed during the apnea maneuver

^a^Post-dive different from pre-dive in the same session

^b^NPB different from PPB

^c^Post-exercise dive different from post-static dive in similar transpulmonary pressure condition

After static dives, Nt-proANP concentrations increased by approximately threefold compared to the pre-dive regardless of breathing pressure (*p* < 0.0001). Nt-proANP concentrations at the end of PPB and NPB exercise dive were respectively five and nine times the pre-dives counterparts (*p* < 0.0001). Conversely, there was no change in Nt-proBNP plasma concentration with any dive.

Dlco and Dlco/Va were unchanged after both static immersions but were significantly reduced after the dives with fin exercise (*p* < 0.0001). Dlco and Dlco/Va were significantly lower after ExNPB compared with ExPPB (*p* < 0.0001).

## Discussion

The study resulted in four important findings. Firstly, we showed that SCUBA diving (immersion) at rest causes a moderate rise in venous return, right heart preload, vascular pulmonary congestion, and ANP release. These findings at rest were independent of breathing pressure. Secondly, exercise combined with PPB breathing increased the cardiovascular effects (i.e., changes in the right heart but not left ventricular indices with the associated right/left heart imbalance) and triggered significant extravascular lung water accumulation thus confirming our previous results [12]. Thirdly, each of these hemodynamic effects as well as the development of interstitial pulmonary edema during exercise was substantially amplified by negative pressure breathing. Fourthly, the cardiovascular changes described correlated with the number of ultrasound lung comet tails representing the degree of extravascular lung water accumulation.

Our findings are important because negative pressure breathing is frequently encountered during SCUBA diving [16, 17, 30, 31]. Diving may increase the effort of breathing due to cold-induced bronchoconstriction, elevated hydrostatic pressure on the chest wall, and resistance of air flow through breathing apparatus [5, 17, 29, 30]. In the present study, tidal volumes and breathing frequency increased during exercise and ventilatory flow rates were reduced to one third of the value expected during exercise on land [22, 29, 30, 32].

Significant small increases in IVC diameter, diastolic right atrial, and ventricle areas were observed during immersion at rest and without substantial difference between the PPB and NPB conditions. The systolic pulmonary artery pressure increased by 80% by immersion alone and is consistent with direct intravascular measures [33]. These changes were compatible with the immersion-induced redistribution of systemic venous blood into the thorax [34, 35]. The compression of limb muscles by external hydrostatic pressure reduces the systemic venous volume, forces venous return, and results in high central venous pressure [33, 36]. A higher central venous pressure results in increased right ventricular contractility via the Frank-Starling mechanism and increases pulmonary artery pressure [37]. A higher pulmonary artery pressure increases capillary hydrostatic pressure and predisposes to the development of interstitial edema [24]. At the end of the dive, the left atrium was enlarged with a corresponding increase in *E*/*A* ratio and decreased EDT, consistent with elevated left ventricle filling pressures secondary to the higher pulmonary artery pressures [24]. After the NPB dive, the increased RV/LV area ratio, an increased TAPSE, and elevated E/A ratio each suggest that a right to left preload imbalance was exacerbated by the negative pressure breathing (Fig. 2) [24].

**Fig. 3:**
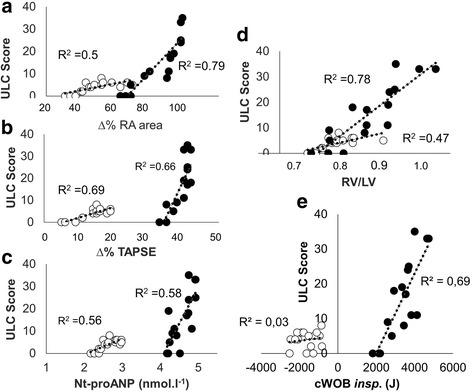
ULC score according to **a** the rise in right atrial area, **b** the rise in TAPSE, **c** the plasma concentration of Nt-proANP, **d** the rise in RV/LV ratio, and **e** the power of breathing, after the exercise dives. ULC score, extravascular lung water score, according to the number of ultrasound lung comet tails. ∆% RA area, percent change from predive in right atrial area; ∆% TAPSE, percent change from predive in tricuspid annular plane systolic excursion; ANP, Nt-proANP plasma concentration at the end of dive; RV/LV, ratio of right to left ventricle end-diastolic area. Empty circles, ExPPB, i.e., setting of positive transpulmonary pressure breathing; full circles, ExNPB, i.e., setting of negative transpulmonary pressure breathing

Heart rate and minute ventilation were increased similarly in both PPB and NPB following dives with exercise. Lung comet tails, however, were much more numerous with NPB compared with PPB (Table 1) and correlated with the imbalance between right and left heart indices (Fig. 3). NPB substantially amplified the hemodynamic changes caused by exercise, and these changes also correlated with accumulation of extravascular lung water. Exercise increased all the indices linked to venous return such as the diameter of inferior vena cava and areas of both right heart chambers. In addition, tricuspid annular displacement and right ventricular fractional area change were increased indicating an increased right ventricle contractility and stroke volume. In contrast, left atrial cross-sectional area increased only mildly and without changes in left ventricular end-diastolic area or stroke volume. The unchanged left ventricular dimensions concomitant with a markedly enlarged right heart are consistent with a picture of relative insufficient left heart output despite the increased right heart preload. In such a scheme, left atrial pressure would be increased because of an increased right ventricular contractility. Indeed, changes in *E*/*A* ratio and EDT displayed a pattern of rapid early filling of left ventricle, indicating increased left atrial pressure with effort, findings which were more marked in the NPB group. Differential changes of right and left cardiac indices suggest an important right to left stroke volume mismatch and an associated increase in pulmonary capillary pressure [12]. The large right heart volume may also limit left ventricular volume within the pericardial sack and exacerbate the right to left stroke volume mismatch (ventricular-ventricular interdependence). In a study by Marabotti et al., *E*/*A* values were higher and EDT was lower during SCUBA breathing at 10 and 5 m depth than pre- and post-dive in air and described by the authors as “typical of restrictive left ventricular diastolic dysfunction” [38]. Their observations are consistent with our results.

Plasma noradrenaline levels were increased by exercise in both the PPB and NPB groups. These changes were similar to changes seen with exercise in other studies [39, 40]. The increases in plasma ANP levels in the NPB were almost double of the values in PPB group. Such high plasma ANP levels have not been reported previously in healthy subjects during exercising in water or during maximal exercising levels on land [40–43]. The levels of ANP probably resulted from the unusually high degree of atrial stretching through the combined effects of (i) immersion, (ii) exercise, and (iii) negative pressure breathing. Interestingly, plasma BNP did not change possibly because there was a no increase in left ventricle volumes or the exercise duration was sufficient [44, 45].

Plasma ANP levels correlated with the lung comet tail score in both exercise sessions (Fig. 3). The elevated levels of ANP during exercise may have exacerbated EVLW accumulation by increasing capillary permeability [27] or by impeding the lymphatic collection of interstitial fluid [46] consequently limiting the removal of interstitial fluid. Finally, the high pressures in vena cava may also limit pulmonary lymphatic flow from the lymphatic duct [47].

Pulmonary edema due to negative pressure on land may develop when a high inspiratory effort generates large negative intrathoracic and alveolar pressure [23]. A more negative intra-thoracic pressure increases the dimension of right atria and ventricle, resulting in a fall in pressure (increasing the vena cava to right atrial pressure gradient), creating an increase in blood volume returning to the right heart [48] and right ventricular contractility through the Frank-Starling mechanism [37]. The combination of a higher pulmonary capillary hydrostatic pressure and a lower lung interstitial pressure promotes plasma fluid extravasation initially into interstitial tissues and then across the alveolar membrane into the alveolar air space [37, 49].

**Fig. 4:**
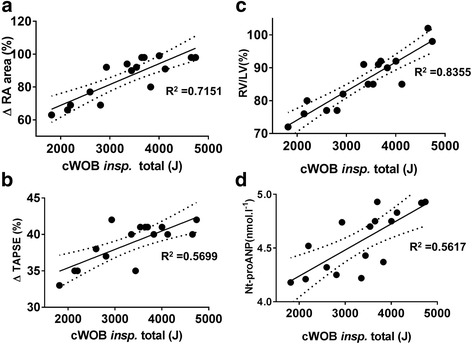
Correlations observed between individual cumulated inspiratory work of breathing and the corresponding percent changes in right atrial volume (**a**), rise in TAPSE (**b**), in RV/LV ratio (**c**), and the final plasma Nt-proANP concentration (**d**), during the fin exercise dive with negative transpulmonary pressure. cWOB insp, cumulated inspiratory work of breathing; ∆RA area, change in right atrial area; ∆TAPSE, change in tricuspid annular plane systolic excursion; RV/LV, ratio of right to left ventricles end-diastolic area; Nt-proANP, plasma concentration of Nt-proANP

We found that combining exercise with negative pressure breathing produced the highest values of TAPSE and mitral *E*/*A* ratio. The cumulated inspiratory work of breathing was strongly correlated with right atria area, plasma ANP concentration, the TAPSE, and the RV/LV area ratio (Fig. 4), i.e., the variables strongly linked to right heart preload.

After the NPB dive with exercise, left atrial volume was only moderately increased but LVEF and SV had not changed from pre-exercise. The RV/LV ratio was also strongly correlated with the cumulated work of breathing (Fig. 4). Recently, we suggested that a discrepancy between the current stroke volumes in the two sides of the heart would cause acute pulmonary edema [9, 24]. At higher heart rates, a relatively small mismatch between right and left ventricular stroke volumes will create extravascular lung water and pulmonary edema because the volume mismatch per minute is increased substantially.

Diving while prone leads to positive pressure breathing; however, on assuming an upright posture, for example, while surfacing, the breathing pressure becomes more negative thus increasing the risk of developing interstitial edema. Extending the duration and intensity of exercise also increases the risk of developing interstitial pulmonary edema (e.g., triathlon) [9, 50]. Some authors have suggested genetic variants may lead to vascular susceptibility to pulmonary edema [51]. The correlations displayed in Figs. 3 and 4, however, clearly show the direct coupling between inspiratory effort, components of heart function, and lung comet score in fit and SCUBA-trained healthy men.

According to the mechanism outlined in this study, any undocumented left heart disease could promote the occurrence of immersed pulmonary edema [4]. Excessive catecholamine release may also cause a stress cardiomyopathy [52]. A higher systemic vascular resistance, as commonly occurs in essential hypertension, may also reduce the ability of the left ventricular stroke volume to increase with effort. The limited increase in left ventricular stroke volume combined with an unhindered increase in right ventricular stroke volume with exercise would promote the development of immersion-induced pulmonary edema. The higher heart rates of exercise, furthermore, exacerbate the ventricular imbalance by increasing the fluid extravasation each minute [24].

The clinical assessment of divers should carefully consider the predisposing and precipitating factors such as left heart disease and hypertension. Similarly, in an individual who has had a previous episode of immersion-induced pulmonary edema, careful considerations surrounding the event and an appropriate cardiac evaluation should be undertaken before resuming diving to prevent recurrences.

### Limitations

The study only included 16 individuals but because of the crossover design, with each individual examined in eight different conditions, we were able to produce highly statistically significant results. The study was not blinded to the participants or ultrasonographer but was analyzed in a blind fashion by an independent researcher. Our study did not determine the effects of depth of a dive; the effects of depth may be important because the gas density is an important determinant of breathing work [29, 30, 32]. Scores of lung comet tails have also been found to be increased at the end of apnea dives either at depth or close to surface when “struggling” inspiratory efforts developed [53]. In the latter study, a 50-m dive apnea caused compressive reduction of lung gas volume and a very large increase in thoracic blood volume. Diaphragmatic contractions during free diving cause lowering of airways and mediastinal pressure similar to the negative transpulmonary pressure breathing seen in our study. It can be surmised that the markedly larger increase in lung comet score found in our study compared with in the apnea diving study was due to the combination of several hemodynamic consequences of negative transpulmonary pressure with larger tidal volumes and of a longer duration. We only investigated men; we are not able to comment on the effects in women. We were unable to determine the independent effects of natriuretic peptides. Right ventricular fraction area change was used instead of the ejection fraction because of the difficulties in calculating the latter by echocardiography. We did not look for the presence of patent foramen ovale in our subjects despite its hypothetical protection from pulmonary edema.

## Conclusions

This is the first study, to our knowledge, to assess the specific impact of exercise on hemodynamics, cardiac function, and effect of breathing pressures in divers. Our study showed that immersion at rest causes modest increases in right heart preload, pulmonary artery pressures, and an imbalance in right and left ventricular physiology but without the development of interstitial pulmonary edema. Negative pressure breathing combined with exercise resulted in much greater increases in right heart preload, pulmonary artery systolic pressure, a greater ventricular mismatch, and worsening interstitial edema. The changes in right heart preload, right to left ventricular imbalance, tricuspid annulus displacement, and pulmonary artery systolic pressures each correlated with the lung comet score. Positive pressure breathing diminishes the cardiovascular changes and decreases the development of interstitial pulmonary edema during effort.

We demonstrated that physically fit young and healthy male divers frequently develop interstitial pulmonary edema during exercise particularly while breathing at a negative pressure. Demonstrating the important influence of breathing pressure on cardiac function during immersed activities has significant implications for preventing the potentially catastrophic condition of immersion-induced pulmonary edema and drowning. The study also highlights the central role of the right ventricle and a right heart-left heart mismatch in generating acute pulmonary edema in cardiovascular disorders [24].
